# Green Synthesized Zinc Oxide Nanoparticles Based on *Cestrum diurnum* L. of Potential Antiviral Activity against Human Corona 229-E Virus

**DOI:** 10.3390/molecules28010266

**Published:** 2022-12-28

**Authors:** Ibrahim N. Alrabayah, Seham S. Elhawary, Zeinab A. Kandil, Essam M. Abd El-Kadder, Yasmine S. Moemen, Abdulrahman M. Saleh, Mohamed A. El Raey

**Affiliations:** 1 Pharmacognosy Department, Faculty of Pharmacy, Cairo University, Giza 11562, Egypt; 2 Trees Research Department, Horticulture Research Institute, Agriculture Research Center, Giza 12619, Egypt; 3 Clinical Pathology Department, National Liver Institute, Menoufia University, Menoufia 32511, Egypt; 4 Pharmaceutical Medicinal Chemistry & Drug Design Department, Faculty of Pharmacy (Boys), Al-Azhar University, Cairo 71524, Egypt; 5 Phytochemistry and Plant Systematics, National Research Centre, Dokki, Cairo 12622, Egypt

**Keywords:** *Cestrum diurnum* L., antiviral activity, coronaviruses, human corona 229E, zinc oxide nanoparticles, HPLC quantification

## Abstract

SARS-CoV-2 has caused more than 596 million infections and 6 million fatalities globally. Looking for urgent medication for prevention, treatment, and rehabilitation is obligatory. Plant extracts and green synthesized nanoparticles have numerous biological activities, including antiviral activity. HPLC analysis of *C. dirnum* L. leaf extract showed that catechin, ferulic acid, chlorogenic acid, and syringic acid were the most major compounds, with concentrations of 1425.16, 1004.68, 207.46, and 158.95 µg/g, respectively. Zinc nanoparticles were biosynthesized using zinc acetate and *C. dirnum* extract. TEM analysis revealed that the particle size of ZnO-NPs varied between 3.406 and 4.857 nm. An XRD study showed the existence of hexagonal crystals of ZnO-NPs with an average size of 12.11 nm. Both ZnO-NPs (IC_50_ = 7.01 and CC_50_ = 145.77) and *C. dirnum* L. extract (IC_50_ = 61.15 and CC_50_ = 145.87 µg/mL) showed antiviral activity against HCOV-229E, but their combination (IC_50_ = 2.41 and CC_50_ = 179.23) showed higher activity than both. Molecular docking was used to investigate the affinity of some metabolites against the HCOV-229E main protease. Chlorogenic acid, solanidine, and catchin showed high affinity (−7.13, −6.95, and −6.52), compared to the ligand MDP (−5.66 Kcal/mol). *Cestrum dinurum* extract and ZnO-NPs combination should be subjected to further studies to be used as an antiviral drug.

## 1. Introduction

Coronaviruses are enveloped, positive-sense m-RNAs that include the most significant known RNA-s genomes with a length of up to 32 kb. Their natural animal hosts have given rise to dangerous variants that threaten human society [1]. Coronaviruses were discovered in 1965 [2]. And more than 30 more strains have been found since then. Standard tissue culture was used to isolate the prototypic stain HCoV-229E (called after a student specimen designated 229E) [3]. Coronavirus strain 229E has been linked to a wide range of respiratory diseases, from the common cold to severe pneumonia [4].

On 31 December 2019, hospitals in Wuhan, China, reported some novel and unusual pneumonia cases with unclear causes. They have been regarded as the essential difficulties a person has encountered in the previous few decades. Researchers used next-generation sequencing and real-time reverse transcription polymerase chain reaction to determine the arrival of a new form of coronavirus known as SARS-CoV2 or COVID-19 (RT-PCR) [5]. The COVID-19 epidemic has escalated into a global crisis. Due to its rapid spread and high fatality rate, SARS-CoV2 is quickly spreading worldwide. Patients with COVID-19 can develop pneumonia, severe acute respiratory distress syndrome (ARDS) symptoms, and multiple organ failure [6]. There have been 596,119,505 infections of COVID-19 reported to WHO as of 5:51 p.m. CEST, 25 August 2022, with 6,457,101 fatalities [7]. Since declaring COVID-19 a global pandemic, researchers have been looking for pharmaceuticals and traditional, alternative, and integrative therapies to aid in the disease’s prevention, treatment, and recovery. Natural medicines are rich sources of substances that may be utilized to prevent and cure a variety of illnesses. Furthermore, natural chemicals have frequently acted as less expensive and safer medication options for a variety of ailments, and several natural substances have been identified as antiviral agents. The number of novel antiviral medications derived from natural sources has increased dramatically during the previous decade. Natural ingredients, either directly or indirectly, are used to find antiviral drugs [8].

Nanotechnology is a rapidly developing field that involves the use of nanoparticles in a variety of applications. The use of nanotechnology in medicine is based on the natural scale of biological processes to develop precise disease prevention, diagnosis, and treatment methods. Nanoparticles offer hope for the development of vaccines and treatments for COVID-19 and other hard-to-treat viral epidemic diseases [9].

The use of inorganic nanoparticles (NPs) for a variety of applications has fueled the demand for green chemical methods for manufacturing. Plants have emerged as the ideal choice for large-scale nanoparticle biosynthesis due to their rapid synthesis rates and the diversity of nanoparticle form and size [10]. Until now, there has been no viable cure for the COVID-19 pandemic. In the fight against COVID-19, nanoparticles (NPs) should be evaluated for use. Their small size and unique features make them helpful in medicinal applications [11].

According to earlier research, ZnO-NPs have been effectively synthesized from various natural extracts, including *Aloe vera*, *Passiflora caerulea*, and *Azadirachta indica*. Furthermore, ZnO-NPs have attracted much interest and are now used in various industries, such as packaging and food additives. ZnO-NPs, unlike other forms of nanoparticles, are easily absorbed by biological tissues and have better biocompatibility with human cells than zinc metal. ZnO-NPs also have antiviral action against various viruses, such as SARS-CoV-2, many respiratory viruses, and herpes viruses [12]. Plant extracts have recently been used to make green ZnO nanoparticles, as they are time-saving, cost-effective, and ecologically benign [13]. ZnO-nanoparticles’ antibacterial efficacy and safety in many industries, such as food, medicine, agriculture, and cosmetics, are outstanding [14]. The US Food and Drug Administration considers ZnO generally recognized as a safe (GRAS) substance [15]. Zinc oxide nanoparticles are used in functional foods and supplements because zinc is important for maintaining human health. ZnO-NPs are also used in food packaging because of their antimicrobial properties [16] and to protect foods from the negative effect of ultraviolet light [17]. ZnO-NPs are used as a component of animal feeds instead of conventional zinc sources because they improve growth rate, immunity, milk production, and reproduction [18].

Antiviral action of ZnO-NPs is mediated by several mechanisms, including the suppression of viral entrance, replication, and dissemination to organs, all of which can result in reactive oxygen species, oxidative damage, and viral death. Zinc-containing compounds showed antiviral efficacy against various viruses through various mechanisms, including physical processes such as virus adhesion, suppression of virus infection, and uncoating. Inhibition of viral polymerases and protease enzymes was also found to demonstrate the action of these compounds [12].

HPLC standardization was a target in our study to investigate the phenolic and alkaloid constituents of *C. diurnum* L. leaves alcoholic extract.

Saponins, lignans, flavonoids, phenolic compounds, volatile oils, and alkaloids have been discovered in the genus Cestrum [19].

The genus showed noticeable biological activities, including: pesticidal, insecticidal, antimicrobial, cytotoxic, hepatoprotective, anticonvulsant, antidiabetic, larvicidal, anti-inflammatory, analgesic, anti-tumor, wound healing, and anti-viral [20].

Cestrum is a genus of around 300 species in the Solanaceae family. One is *Cestrum diurnum* L., a multi-stemmed shrub frequently known as “Day Jasmine.” [21].

*C. diurnum* L. was reported to contain saponins [22], flavonoids [23], and lignans [24]. These compounds were noted for their critical biological activities, such as anti-inflammatory [25], antimicrobial [26], cytotoxic, thrombolytic [27], and antiviral activities [28,29]

Molecular modeling is one of the fundamental ways to simulate and picture how a target site is likely to bind based on specific features [30].

The aim of this study is to green-synthesize ZnO-NPs based on the leaf alcoholic extract of *C. diurnum* L. It examines the antiviral activity of the alcoholic extract, ZnO-nanoparticles, and their combination against human corona-229E, discovering the mode of action and predicting the mechanism of action using molecular docking simulations.

## 2. Results and Discussion

### 2.1. HPLC Analysis of Phenolic Constituents

**Figure 1:**
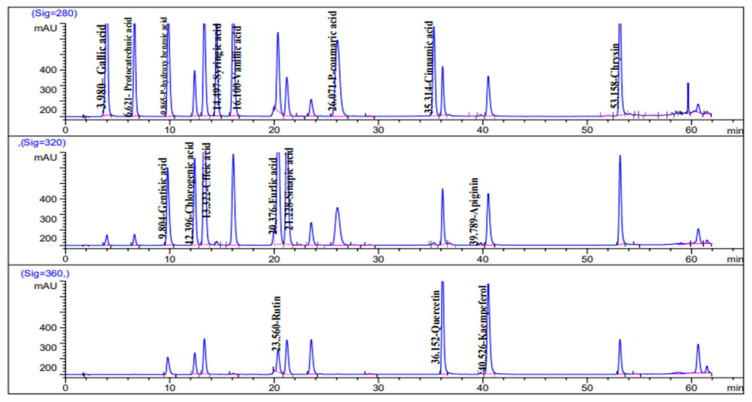
HPLC chromatogram of phenolic compounds (standard mixture).

**Figure 2:**
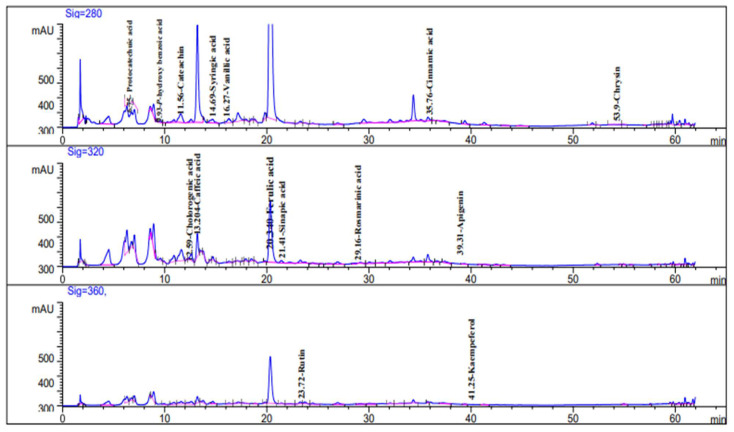
HPLC chromatogram of phenolic compounds of *C. diurnum* leaves extract.

HPLC investigation of *C. diurnum* L. leaf alcoholic extract (Figure 1 and Figure 2) revealed the presence of 15 components. Catechin, ferulic acid, chlorogenic acid, and syringic acid were the main components, with concentrations of 1425.16, 1004.68, 207.46, and 158.95 μg/g, respectively. On the other hand, protocatechuic acid, *P*-hydroxy benzoic acid benzoic acid, caffeic acid, vanillic acid, sinapic acid, rutin, rosmarinic acid, cinnamic acid, apigenin, kaempeferol, and chrysin were detected in lower concentrations (Table 1). Rutin was previously detected [31,32] in the flower extract of *C. elegans* and the leaves of *C. nocturnum*. Caffeic acid ester has been detected in *C. euanthes* [33]. Chlorogenic acid has been previously detected in *C. elegans* and *C. poepigii* [19].

### 2.2. HPLC Analysis of Alkaloid Constituents

**Figure 3:**
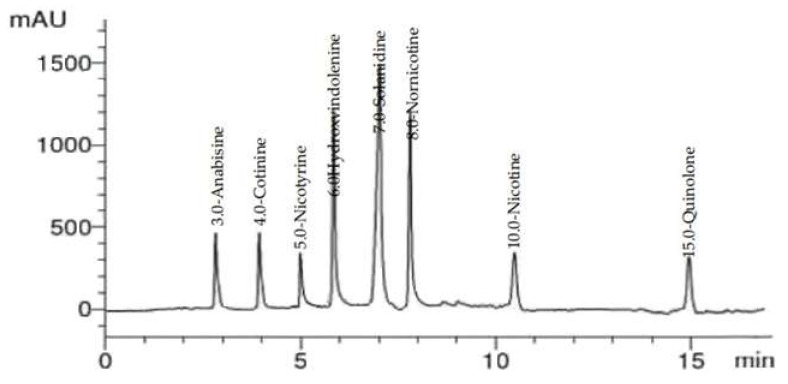
HPLC chromatogram of alkaloid compounds (standard mixture).

**Figure 4:**
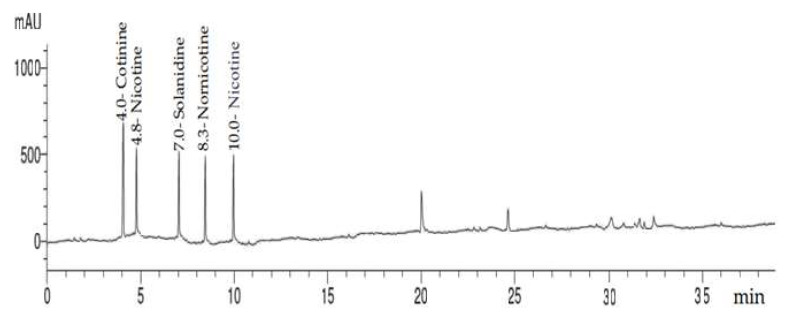
HPLC chromatogram of *C. diurnum* leaf parts (ethanolic extract).

HPLC analysis of *C. diurnum* L. leaves alcoholic extract (Figure 3 and Figure 4) indicated the presence of cotinine, nicotyrine, solanodine, nornicotine, and nicotine with concentrations of 10.66, 9.74, 9.65, 8.89, and 8.55 µg/g, respectively (Table 2). Solanidine was previously detected in the leaves of *C. purpureum* [19]. Nicotine and nornicotine were detected in *C. diurnum* and *C. nocturnum* leaf extracts, and cotinine was detected in *C. nocturnum* leaf extract [34].

### 2.3. Characterization of ZnO-Nanoparticles

#### 2.3.1. UV Analysis

The highest absorption peak for ZnO-NPs produced with *C. diurnum* L. leaf extract was at 278, 365 nm (Figure S1, Supplementary Materials), indicating that ZnO-NPs had been formed. Compared to the typical ZnO absorption pattern, shorter wavelengths are found in nanoscale ZnO, which is consistent with claims that material oxides and nanoscale materials have shorter wavelengths [8].

#### 2.3.2. FT-IR Analysis of ZnO-NPs and *C. diurnum* L. Leaf Extract

**Figure 5:**
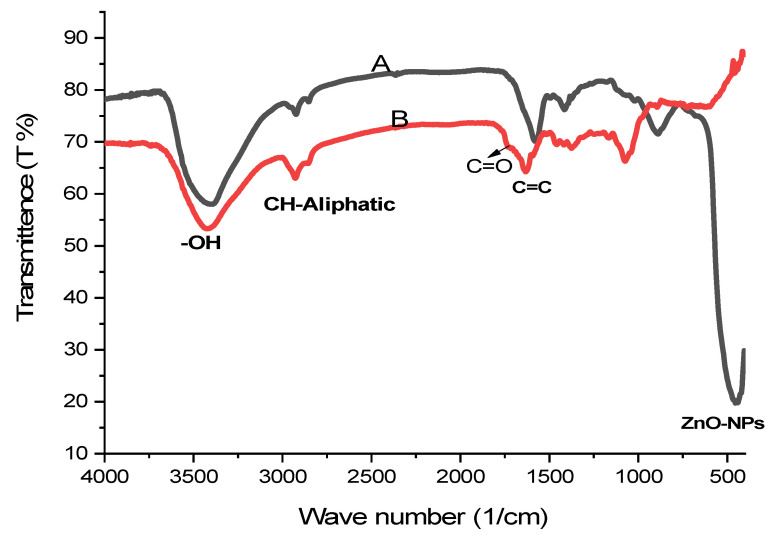
FT-IR spectrum of alcoholic extract of the *C. diurnum* leaf parts (ethanolic extract) (**A**), and FT-IR spectrum of ZnO-NPs (**B**).

In order to determine the different involved functional groups, FT-IR spectra of ZnO-NPs and *C. diurnum* L. leaf extract were investigated in the region of 400 to 4000 cm^−1^ wavelength. The ZnO-NP spectrum in Figure 5A shows a characteristic absorption band at 3398 cm^−1^, indicating the water’s OH group adsorbed on the ZnO-NPs surface. Bands at 2924 and 2854 cm^−1^ are indicated for the C-H stretching of alkane groups. The bands at 1122 and 1026 cm^−1^ indicated C-O of ethers, carboxylic acid esters, or alcohols. The emergence of a band at 887.26 confirms that the ZnO-NPs underwent C-H bending, and the characteristic band of the ZnO-NPs stretching mode was assigned to 443.63 [8]. Figure 5B: *C. diurnum* L. extract revealed an essential broadband peak at 3425 cm^−1^, confirming the presence of a carboxylic OH, phenolic, or alcoholic hydroxyl-OH group. The absorption band at lower wavenumbers of 1600 to 1700 cm^−1^ of C=O (carboxylic and ketonic) is a result of delocalization of its π electrons due to conjugation with C=C in both flavonoids and carboxylic acid compounds [35]. The peaks observed at 2927 and 2854 cm^−1^ were assigned to the C-H stretching of the alkane group. The presence of bands at 1631–1377 cm^−1^ was assigned for C=C or C-C stretching of polyphenolic compounds. A band at 1072 cm^−1^ indicated the bending vibration of C-H in alkane groups. The presence of moderate bands at 891–894 cm^−1^ was assigned to the alkene (=C-H) groups. The peak at 1037 cm^−1^ revealed the existence of the C-H alkane group.

#### 2.3.3. Light Scattering Dynamics and Zeta Potential

**Figure 6:**
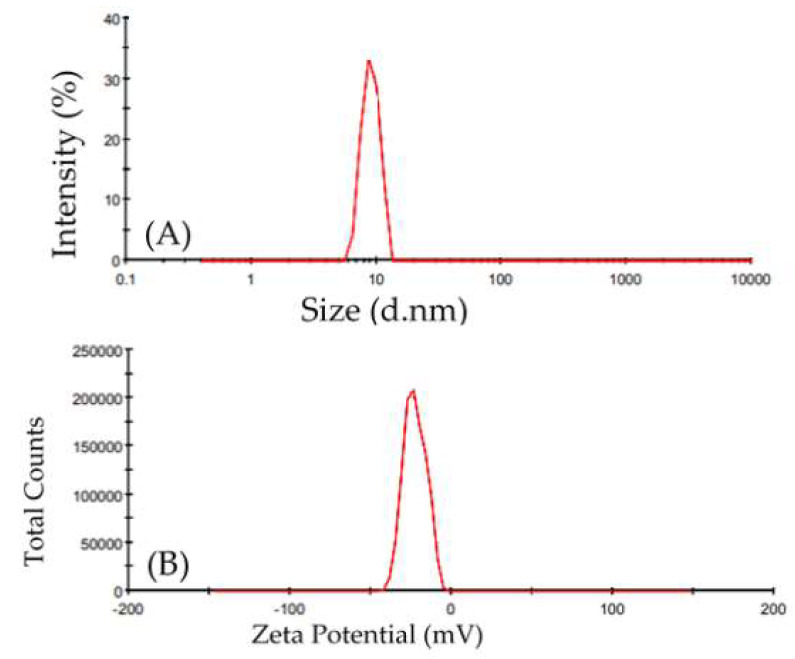
(**A**) Zeta size of nanoparticles synthesized of zinc oxide (Z-average (d.nm): 8.903, PdI: 0.242, and intercept: 0.88), (**B**) Zeta potential of zinc oxide nanoparticles produced via green synthesis (Zeta potential (mV): −22.6, Zeta deviation (mV): 6.9, and conductivity (mS/cm): 0.0113).

The size-distribution image (DLS) of the green-synthesized nano-ZnO is shown in Figure 6A. The average particle size distribution of NPs was 8.903 nm, with a PDI value of 0.242. The zeta potential of ZnO-NPs gives information about nanoparticle stability. It was shown to peak at −22.6 mV (Figure 6B), indicating that the biosynthesized nano-ZnO particles were negatively charged and dispersed evenly throughout the media. The stability of the nanoparticles was caused by negative values determined by the zeta potential [10]. Regarding the zeta potential and zeta sizer of the combination (Figure S2, Supplementary Materials), the combination showed a peak at −25.1 mV, which reflects the greater stability of the combination than nano-ZnO particles. This enhanced effect of stabilization is a result of the capping agent [36].

#### 2.3.4. Transmission Electron Microscope (TEM) and Scanning Electron Microscopy (SEM) Analysis

**Figure 7:**
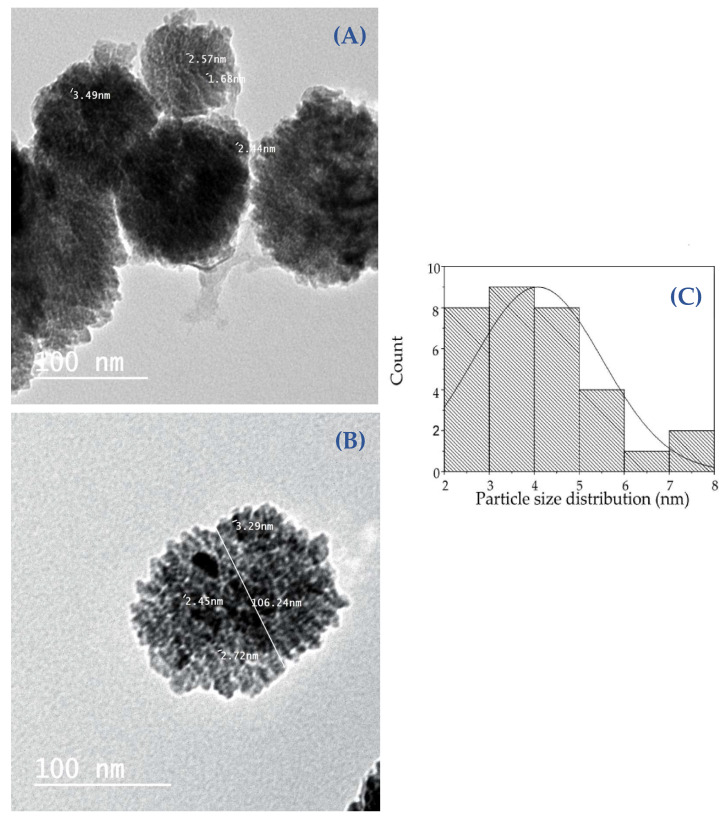
TEM image of green-synthesized Zno-NPs (**A**,**B**) and a histogram of the particle size distribution (**C**).

**Figure 8:**
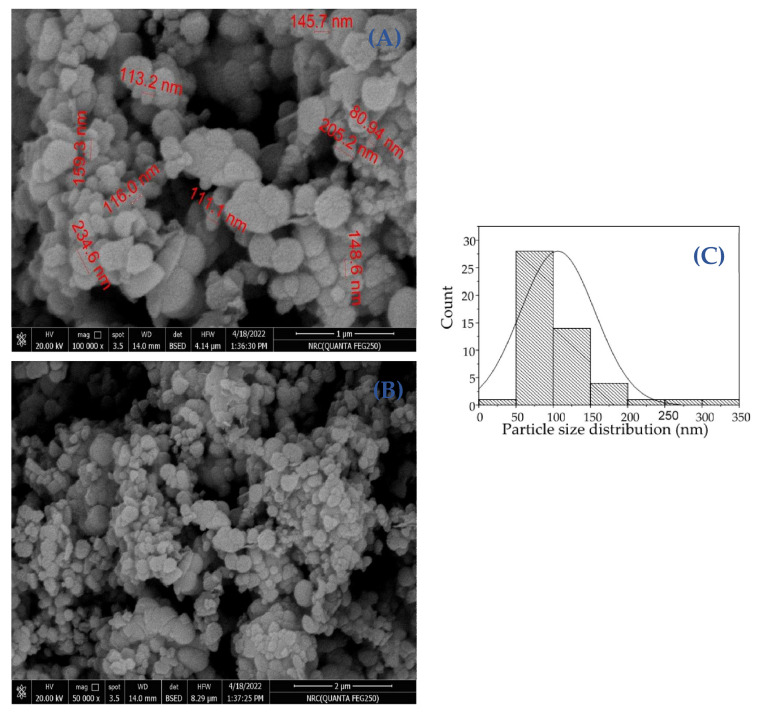
ZnO-NPs SEM image (**A**,**B**) and a histogram of the particle size distribution (**C**).

The TEM study was conducted to learn more about the ZnO-NPs’ nature and crystallinity. The particles were massive hexagonal crystals measuring between 3.406 and 4.857 nm with a mean of 4.079 ± 1.437, as seen in Figure 7A–C. The morphological structure of green nanoparticles was investigated using SEM analysis (Figure 8A,B). The structure of the particles is spherical. The particle size of the ZnO-NPs nanoparticles ranges from 80.94 to 234.6 nm, with a range of 106 ± 4.5 (Figure 8C). The homogeneous distribution of the particles well describes the nature of the particles’ sizes. Previous studies have shown that particle agglomeration occurs in nanoparticles that employ natural products as reducing and capping agents, and the particles seem to have bigger particle sizes [37].

#### 2.3.5. X-ray Diffraction (XRD) Analysis

**Figure 9:**
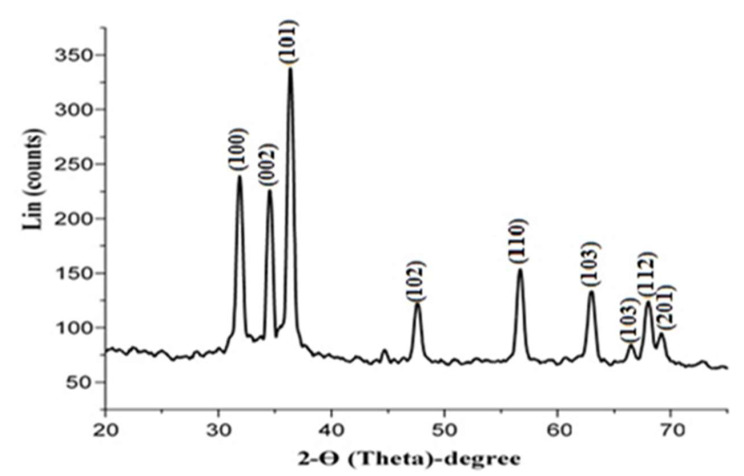
XRD analysis of ZnO-NPs.

X-ray diffraction was used to demonstrate the presence of nano-ZnO and to investigate its structural features (XRD). Nano-ZnO bio-synthesized *C. diurnum* extract exhibited peaks with 2θ (theta) values fully recognized at 31.88143°, 34.54302°, 36.36804°, 47.61228°, 56.67371°, 62.96601°, 62.97003°, 68.02982°, and 69.01234° corresponding to (100), (002), (101), (102), (110), (103), (103), (112), and (201), respectively (Figure 9). These peaks were matched with those of the data card (00-003-0888). Scherrer’s equation calculated the average ZnO-NPs crystal size to be 11.16 nm.

### 2.4. Antiviral Activity

Although there is a lack of information about the antiviral activity of zinc oxide nanoparticles against the human corona 229 E virus, there have only been two very recent reports about this issue. Alqahtani et al., 2022, studied the antiviral activity of Pelargonium zonale extract, green synthesized nanoparticles, and their combination against human corona 229E and showed that their combination was the most bioactive, and it has a selective index of 68 [38]. On the other hand, AbouAitah et al., 2022, studied the same activity using pure ellagic acid, ZnONPs, and their combination and showed also that the combination has a selective index of 75 [39].

Table 3 revealed that both ZnO-NPs and the total alcoholic extract of *C. diurnum* L. leaf extract exhibited significant antiviral activity against human corona 229E. However, their combination had more vigorous activity than the ZnO-NPs and total alcoholic extract themselves, with selective indexes of 74.37, 20.81, and 3.9, respectively. Selective index (SI) = estimated CC_50_/estimated IC_50_. The selective index of our combination is similar to the combination of ellagic acid with nano-ZnO. These findings refer to the presence of potential anti-human coronavirus 229E compounds in the extract. This suggestion was discussed by molecular docking simulation.

The tested samples were described as good candidates for further experiments as anti-coronaviruses.

The antiviral activity of *C. diurnum* may be attributed to the different identified phytoconstituents via HPLC analysis. The obtained results follow the previously published features regarding the antiviral activities of these phytoconstituents. Caffeic acid has previously been reported to have antiviral activities against COVID-19, HIV, herpes simplex, and hepatitis B [40,41,42,43]. Chlorogenic acid has been detected to be antiviral against COVID-19 and influenza A (H1N1/H3N2) viruses [44,45]. Furthermore, cinnamic acid has been shown to have anti-herpes and anti-Zika virus properties [46,47]. Rosmarinic acid, rutin, and sinapic acid have been reported for their anti-COVID-19 activities [48,49,50]. Rutin has also been shown to have anti-dengue virus activity [51]. In addition, catechin has been reported for its anti-influenza A (H1N1) activity [52].

Regarding the mode of antiviral activity, (Table 4) revealed that during replication, the combination of ZnO-nanoparticles and leaf extract showed higher antiviral activity against human coronavirus (229E) replication with inhibition activity up to 72.8% than adsorption and virucidal with inhibition activity up to 17.2% and 57%.

**Figure 10:**
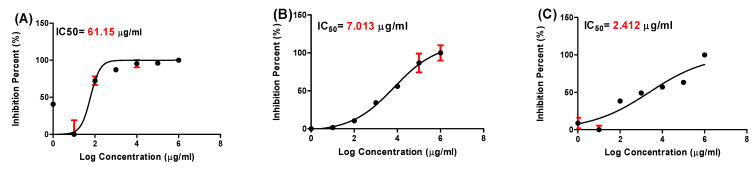
The antiviral effects (IC_50_) of the compounds tested against the corona 229-E virus. Nonlinear regression analysis was used to create each graph. The log concentrations were plotted against the cell viability normalized response (**A**): *C. diurnum L.* leaf extract, (**B**): ZnO-NPs, and (**C**): Combination).

Figure 10 depicts the antiviral activity of each material against the human corona 229-E virus, as evaluated by the CPE. The IC_50_ varied in response, which differs between *C. diurnum* leaf extract, ZnO-NPs, and their combinations. In the case of ZnO-NPs, viral inactivation was greater (lowest IC_50_ 7.013) than the *C. diurnum* L. leaf extract alone (IC_50_ 61.15). Nevertheless, viral inactivation was greater in the case of combinations (IC_50_ 2.412) than in the case of *C. diurnum* L. leaf extract and ZnO-NPs.

### 2.5. In Silico Assessment and Molecular Docking Studies

#### 2.5.1. Docking Studies of Experimental Ligands

**Figure 11:**
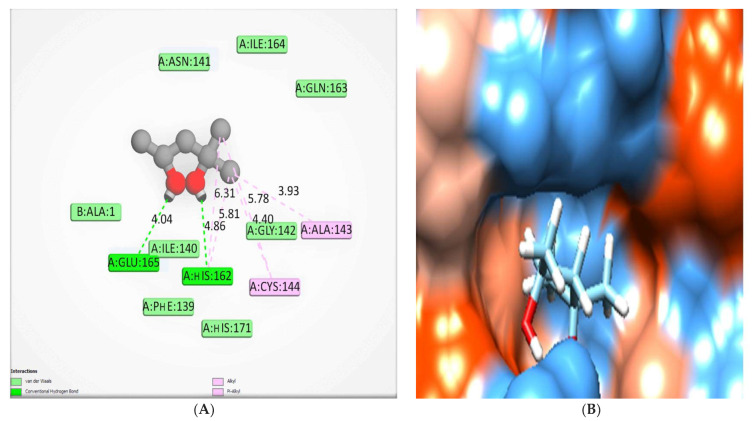
The crystal structure MDP has been re-docked in HCo-V 229E main protease, where hydrogen bonds (green) and pi interactions are represented in pink lines (**A**) with a mapping surface demonstrating the crystal ligand occupying the active pocket of HCo-V229E main protease (**B**) with binding energy −5.66 Kcal/mol.

The crystal structure of HCo-V 229E main protease (2ZU2) contains two ligands, which are zinc (II) hydrogen sulfide (DTZ), H2 S2 Zn, and (4s)-2-methyl-2,4-pentanediol (MDP), C_6_H_14_O_2_, Figure 11 and Table 5.Such ligands interact with chain A only, as in the case of MDP, or interact with both chains A and B, as in the case of DTZ. Here, we used MDP as a molecular docking validation because its size is more convenient to the current molecular docking study.

#### 2.5.2. Docking Studies of Phenolic Compounds

**Figure 12:**
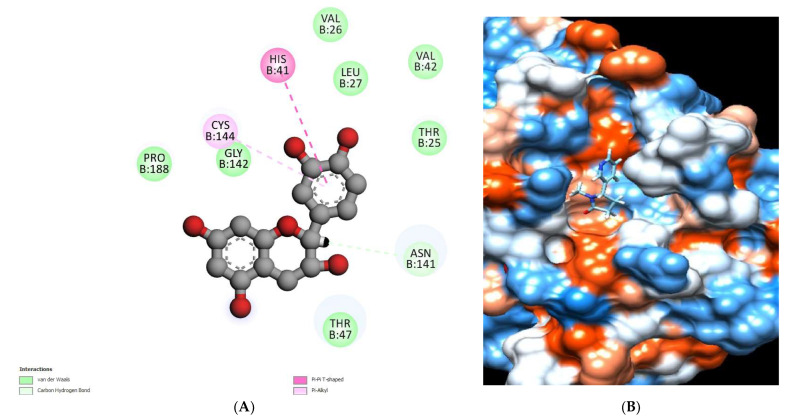
Catechin docked in HCo-V229E main protease, hydrogen bonds (green), and pi interactions are represented in purple lines (**A**), with the mapping surface showing catechin occupying the active pocket of Co-V229E main protease (**B**).

The binding mode of catechin exhibited an energy binding of −6.52 kcal/mol against the HCo-V 229E main protease, which formed Pi-Alkyl and Pi-Pi interactions with His41, ILe164, and Pro188. Additionally, it interacted with Glu165 and Gln191 by two hydrogen bonds with 4.06 and 3 Å, respectively (Figure 12) and Table 5.

**Figure 13:**
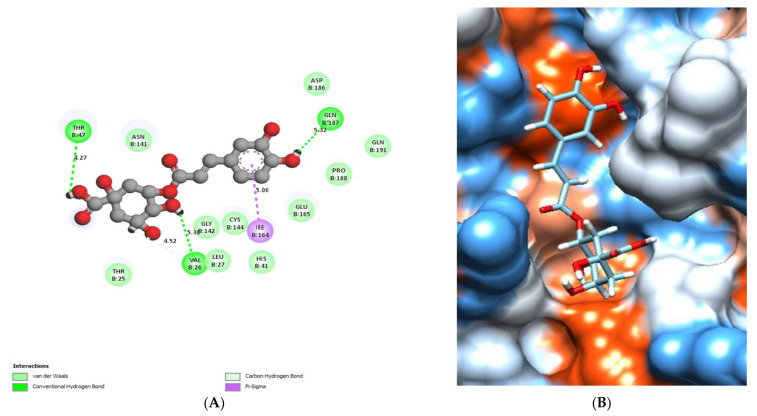
Chlorogenic acid docked in HCo-V229E main protease hydrogen bonds are represented in green lines (**A**), with the mapping surface showing chlorogenic acid occupying the active pocket of HCo-V229E main protease (**B**).

The binding mode of chlorogenic acid exhibited an energy binding of −7.13 kcal/mol against the HCo-V229E main protease target site. Chlorogenic acid interacted with Val26, Thr47, and Gln187 by three hydrogen bonds with 5.38, 4.26, and 5.32 Å (Figure 13) and Table 5.

**Figure 14:**
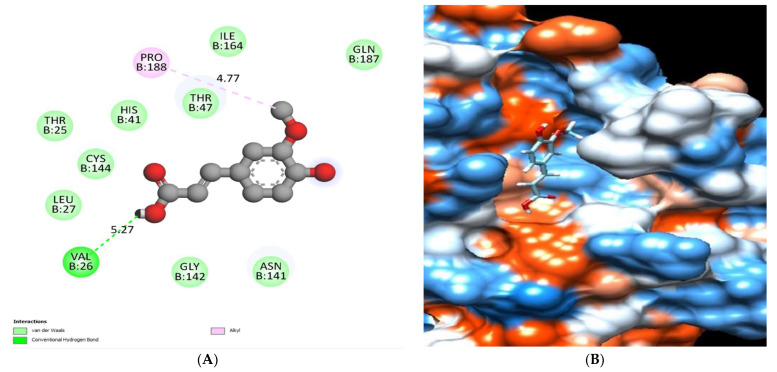
Ferulic acid docked in HCo-V 229E main protease hydrogen bonds are represented in green and the pi interactions are represented in pink lines (**A**), with a mapping surface showing ferulic acid occupying the active pocket of HCo-V 229E main protease (**B**).

The binding mode of ferulic acid exhibited an energy binding of −6.00 kcal/mol against HCo-V 229E main protease. Ferulic acid interacted with Val 26 by a hydrogen bond with a distance of 5.27Å and formed a pi-alkyl interaction with Pro188 (Figure 14) and Table 5.

**Figure 15:**
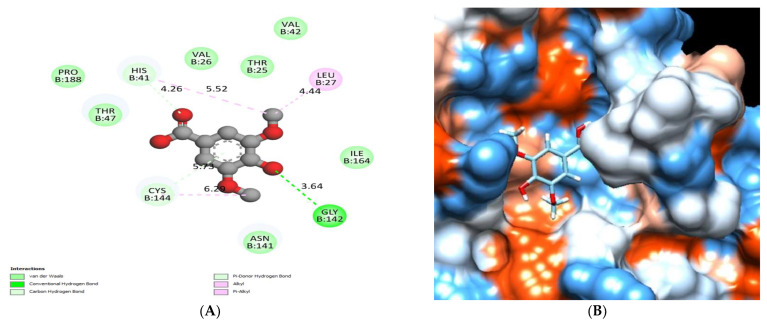
Syringic acid docked in HCo-V 229E main protease. Hydrogen bonds are represented in green and pi interactions are described in pink lines (**A**), with a mapping surface showing syringic acid occupying the active pocket of HCo-V 229E main protease (**B**).

The binding mode of syringic acid exhibited an energy binding of −6.25 kcal/mol against HCo-V 229E main protease. Ferulic acid formed three pi-alkyl interactions with Leu27, His41, and Cys144, which interacted with Gly142 with a hydrogen bond 3.64 Å (Figure 15) and Table 5.

#### 2.5.3. Docking Studies of Alkaloid Compounds

**Figure 16:**
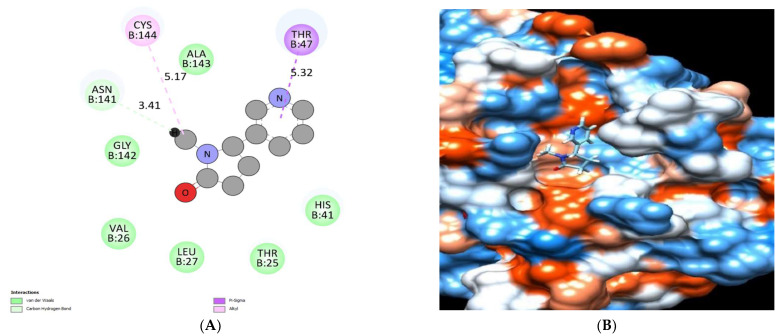
The binding mode of cotinine exhibited an energy binding of −6.12 kcal/mol against HCo-V 229E main protease. Hydrogen bonds are represented in green and pi interactions are described in pink lines (**A**), with a mapping surface showing cotinine occupying the active pocket of HCo-V 229E main protease (**B**).

The binding mode of cotinine with HCo-V229E protease is through forming pi-Sigma interaction with Thr 47. Moreover, it interacted with the Asn141 hydrogen bond at a distance of 3.41 Å (Figure 16) and Table 5.

**Figure 17:**
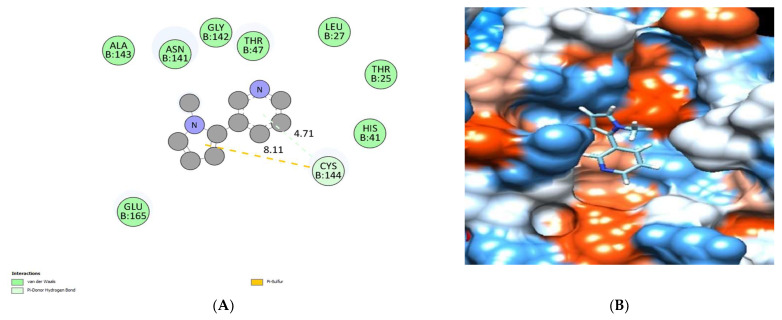
Nicotyrine docked in HCo-V229E main protease. Hydrogen bonds are represented in green, and the pi interactions are described in dark yellow lines (**A**), with a mapping surface showing nicotyrine occupying the active pocket of HCo-V229E main protease (**B**).

The binding mode of the nicotyrine exhibited an energy binding of −5.75 kcal/mol against HCo-V 229E main protease, which formed a pi-Sigma interaction with Cys144. Additionally, it interacted with His41 through one hydrogen bond with a distance of 4.71 Å (Figure 17 and Table 5.

**Figure 18:**
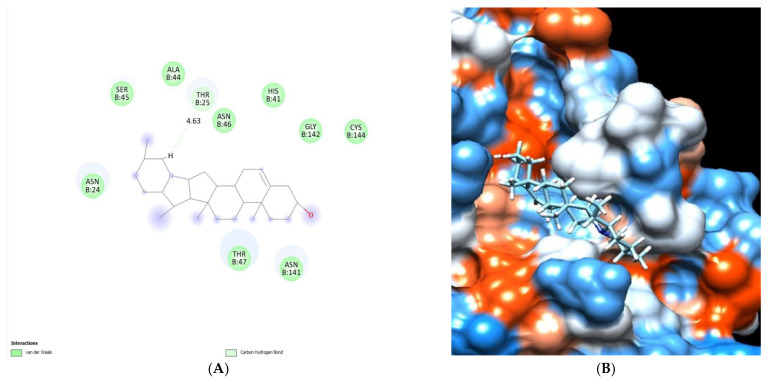
Solanidine docked in HCo-V 229E main protease. Hydrogen bonds are represented in green, and the pi interactions are represented in green lines (**A**), with a mapping surface showing solanidine occupying the active pocket of HCo-V 229E main protease (**B**).

The binding mode of the solanidine exhibited an energy binding of −6.95 kcal/mol against Co-V 229E main protease, which formed a hydrogen bond at Thr 25 with a distance of 4.63 Å (Figure 18) and Table 5.

## 3. Material and Methods

### 3.1. Plant Material

Different organs of *C. diurnum* were collected from El-Orman Botanic Park, Giza, Egypt. The plant was identified by Madam Therease Labib, consultant of plant taxonomy at the El-Orman botanical garden, Giza, Egypt.

The herbarium sample retained at the Department of Pharmacognosy, Faculty of Pharmacy, Cairo University (Giza, Egypt), has a voucher specimen (number: 11112020).

### 3.2. Extraction

A total of 100 g of coarse air-dried powdered leaves of *C. diurnum* were repeatedly macerated in 70% ethanol (250 mlx3). The obtained hydroethanolic extract was evaporated under reduced pressure, yielding 2.5 g of dry extract.

### 3.3. HPLC Analysis of Phenolic Constituents

A liquid chromatography system (Agilent Technologies 1100 series) featuring an autosampler and a photodiode-array detector is used for HPLC analysis. An Eclipse XDB-C18 (4.6 × 150 mm, 5 µm) analytical column with a C18 guard column was used (Phenomenex, Torrance-CA). Acetonitrile (solvent A) and aqueous acetic acid (2%) (Solvent B) were used as mobile phases. The flow rate was held constant at 0.8 mL/min for a total run time of 70 min, and the gradient program was as follows: 100% B to 85% B in 30 min, 85% B to 50% B in 20 min, 50% B to 0% B in 5 min, and 0% B to 100% B in 5 min. The injection volume was 50 µL, and at 280 and 320 nm, the peaks for benzoic acid and cinnamic acid derivatives were examined concurrently. All samples were filtered using a 0.45 m syringe filter before injection (Gelman Laboratory, MI, USA). The peaks were identified using congruent retention times and UV spectra, which were then compared to the standards [53].

### 3.4. HPLC Analysis of Alkaloid Constituents

A Thermo Scientific Accela ultra-performance liquid chromatography (UPLC) system was used for analysis. Aligent Zorbax-C18 USA (2.1 × 150 mm, 1.8 µm) analytical column with a C18 guard column was used (Phenomenex, Torrance, CA). The isocratic mobile phase consisted of 90:10 (*v*/*v*); 20 mM CH_3_COONH_4_/CH_3_CN. For 40 min, the flow rate was kept constant at 1 mL/min. The injection volume was 25 µL, and the standard alkaloids’ peaks (cotinine, nicotyrine, solanodine, nornicotine, and nicotine) were simultaneously monitored at 260 nm. Before injection, all samples were filtered via a 0.45 µm syringe filter (Gelman Laboratory, MI, USA). Congruent retention times and UV spectra were used to identify the peaks, which were then compared to the standards [53].

### 3.5. Green Synthesis of Zinc Oxide Nanoparticles

ZnO-NPs were made using the alcoholic extract of the leaves of *C. diurnum* as a capping, stabilizing, and reducing agent by the technique described by Melk et al., 2021 [10]. Briefly, *C. diurnum* dried leaf extract (1 g) was dissolved in hydroalcohol (100 mL) and then mixed with zinc acetate (5 g), which is dissolved in bi-distilled H_2_O and heated in a water bath for 20 min at 100 °C, then a few drops of ammonium hydroxide were added to keep the pH of the reactant media at 12. Whereby, ZnO-NPs precipitate was formed, then the mixture was allowed to sit for another 20 min at 100 °C to complete the conversion of zinc acetate to ZnO-NPs. After centrifugation at 4000 rpm, the collected ZnO-NPs pellets were washed twice with bi-distilled water and twice with ethanol. Finally, the generated nanoparticle pellets were freeze-dried.

### 3.6. Characterization of Zinc Oxide Nanoparticles

#### 3.6.1. V-Vis Spectral Analysis

A UV spectrophotometer (Shimadzu, UV-1601) was used to evaluate ZnO-NP preparations (Shimadzu Corporation, Kyoto, Japan). Between 200 and 400 nm, UV spectra were recorded. 

#### 3.6.2. FT-IR Analysis

Using a Shimadzu FT-IR Affinity-1 Spectrometer (Shimadzu Corporation, Kyoto, Japan) in attenuated total reflectance mode, the functional groups and other phytochemical compounds responsible for nanoparticles’ production and stabilization were determined. 

#### 3.6.3. Zeta-Sizer Measurements

Dynamic light scattering was performed using a Zeta-sizer Nano-zs laser diffractometer (Malvern, Worcestershire, UK). 

#### 3.6.4. Transmission Electron Microscopy (TEM) and Scanning Electron Microscopy (SEM) Analysis

The shape and particle size of ZnO-NPs mediated by *C. diurnum* extract were investigated using TEM (JEOL-JEM-1011, JEOL Ltd., Tokyo, Japan). 

The emission scanning electron microscope (FESEM, model Quanta 250 field emission gun (FEG), with accelerating voltage of 30 Kv, magnification of 14 to 106 x, and resolution for gun. 1n, FEI Company, USA.

A few droplets of ZnO-NPs dispersion were placed on a carbon-coated copper grid and the solvent was evaporated at room temperature before taking the photographs.

#### 3.6.5. X-ray Diffraction (XRD)

Utilizing a Bruker D8 Advance Diffractometer (Bruker AXS, Karlsruhe, Germany) and Cu Ka radiation (k = 1.54), an X-ray diffraction (XRD) analysis of the produced solid materials was carried out. ZnONPs’ XRD pattern was consistent across a 2-theta range of 10–90. Energy-dispersive x-ray spectroscopy was used to get the data.

### 3.7. Evaluation of the Antiviral Activity

A variety of viruses and cell lines were employed in this study. They were purchased from the American Type Culture Collection (ATCC, Manassas, VA, USA) and kindly provided by Nawah-Scientific Co., Cairo. Several viruses and cell lines were used. For the propagation of human coronavirus-229E, the host cell lines were clones of Vero (Vero-E6) cells and (corona-229E). The cells were grown in DMEM medium-high glucose (Grand Island, NY, USA) supplemented with 10% foetal bovine serum (Grand Island, NY, USA), 0.1 percent antibiotic/antimycotic solution (Gibco BRL, Grand Island, NY, USA), and trypsin-EDTA (Grand Island, NY, USA) [39].

#### 3.7.1. Cytotoxicity Assessment

The crystal violet technique was employed to evaluate the experiment. In brief, Vero E6 cells were seeded into a 96-well culture plate at a density of 2 *×* 104 cells/well one day before infection. The next day, the growth media was withdrawn, and the cells were rinsed in phosphate-buffered saline. 0.01 mL of 70% cold acetone was added to each well. The plate was kept at −20 °C for 30 min. The acetone was removed, and the 96-well plate was oven-dried at 60 degrees Celsius for 30 min. The plate was then incubated with 0.01 mL of 0.4% sulforhodamine B (SRB) (*w*/*v*) in 1% acetic acid (*v*/*v*) at room temperature for 30 min. The plate was washed five times with 1% acetic acid (*v*/*v*) and left to dry to eliminate unbound sulforhodamine B. After that, 100 mL of a 10 mM unbuffered Tris base solution was added to the wells at room temperature and left for 30 min to solubilize the fixed SRB. Finally, the optical density (OD) was determined at 540 nm using a microplate reader (BMG LabTech GmbH FLUOstar Omega, Ortenberg, Germany). The reference absorbance was measured at 620 nm using the Graph Pad PRISM program. The 50% cytotoxic concentration (CC_50_) was calculated (Version 5, GraphPad Software, San Diego, CA, USA) [39].

#### 3.7.2. Antiviral Assessment

The assay was performed using the method of Hayden et al. [46]. Vero E6 cells (the golden cell line for coronavirus propagation and titration) were cultured for 24 h at 37 °C in a six-well plate. In parallel to the untreated viral control, the virus was incubated with varying concentrations of the sample for 30 min. The cells were inoculated with (100 l/well) countable virus/sample mixes after the growth media was withdrawn from the cell culture plates. A total of 1.5 mL of DMEM mixed with 2% agarose was added to the cell monolayer after one hour of contact. Plates were allowed to harden before being incubated at 37 °C until the formation of viral plaques (through 3 days). After two hours of 10% formalin in distilled water, the plates were colored with 0.1% crystal violet. The untreated virus was incubated with Vero E6 cells in control wells, plaques were enumerated, and the plaque reduction% in comparison to control was reported according to the following equation:

Viral count (untreated) − viral count (treated)/viral count (untreated) × 100 = percent inhibition [39]. 

### 3.8. Mode of Action of Antiviral Activity

#### 3.8.1. Adsorption Mechanism

Vero E6 cells were grown for 24 h at 37 °C in a six-well plate. The cells were injected with 100 µL/well of various test substance concentrations of the test substance after the growth media withdrawal from the cell culture plates. The virus (100 µL/well) was injected after a one-hour incubation period. 1.5 mL DMEM mixed with 2% agarose was added to the cell monolayer after one hour of contact. Plates were allowed to harden before being kept at 37 °C until the formation of viral plaques (through 3 days). After two hours of formalin (10%), the plates were dyed with 0.1% crystal violet in distilled water. The untreated virus was incubated with Vero E6 cells in control wells. Plaques were enumerated, and the plaque reduction % in comparison to the control was reported according to the following equation:

Percent inhibition = viral count (untreated) − viral count (treated)/viral count (untreated) × 100 [39].

#### 3.8.2. Replication Mechanism

Vero E6 cells were grown for 24 h at 37 °C in a six-well plate. The virus was injected (100 mL/well) and incubated at 37 °C for one hour after the growth media was withdrawn from the cell culture plates. Then, the infected cells were injected for one hour with various doses of the tested substance (100 µl /well) and incubated at 37 °C. After one hour of contact, 1.5 mL of a mixed DMEM with 2% agarose was added to the cell monolayer. Plates were allowed to harden and then incubated at 37 °C until viral plaques were formed (through 3 days). After two hours with 10% formalin, the plates were dyed with 0.1% crystal violet in distilled H_2_O. Untreated virus was incubated with Vero E6 cells in control wells, and plaques were enumerated. The plaque reduction % in comparison to the control was reported according to the following equation:

Viral count (untreated) − viral count (treated)/viral count (untreated) x 100 = percent inhibition [39].

### 3.9. In Silico Assessment and Molecular Docking Studies

Some identified metabolites from *C. diurnum* were screened against HCOV 229-E 3CL protease target sites to predict the expected target that is affected by our natural products, using the crystal protein structure, where the binding sites were created by co-crystallizing the ligand with the crystal protein (PDB code: 2ZU2) [54]. Using SwissDock, some processes should have been performed to provide insight into the molecular binding modes of the tested compound inside the pockets of Co-V 229E main protease.

SwissDock is a docking application modelled after EADock DSS [55]. The following procedures make up its algorithm. Starting with a large number of BMs (usually between 5000 and 15,000), either in a user-defined box (local docking) or close to the target cavities of the entire protein surface, is performed (blind docking). Meanwhile, their CHARMM [56] energies are estimated on a grid. Afterwards, BMs with the best energies are prioritized and clustered using the FACTS implicit solvation model [57], which considers the solvent effect.

Last but not least, the optimal clusters are written to the output file. Due to this one-of-a-kind set of characteristics, precise docking assays can be performed in a matter of minutes. UCSF Chimera package and Discovery Studio Visualizer v21.1 [58] were used for protein binding mode demonstration.

### 3.10. Statistical Analysis

The GraphPad PRISM software V.5 was utilized for computations and statistical analysis. The mean and standard deviations (SDs) were calculated for each experiment. The significance between the tested agents and the positive control was determined using ANOVA and Tukey’s multiple comparison test.

## 4. Conclusions

HPLC standardization showed that *C. diurnum* is rich in both phenolic and alkaloid metabolites. The alcoholic extract of *C. diurnum* was effectively green-synthesized ZnO-NPs. The FT-IR analysis results showed that the surface of ZnO nanoparticles had many adsorbed functional groups. The production of zinc oxide nanoparticles is further supported by FT-IR spectroscopy. In this study, we showed for the first time the environmentally friendly manufacturing of ZnO-nanoparticles using *C. diurnum* L. and their anti-HCOV-229E potential.

The antiviral activity of the ZnO-NPs and their combination with *C. diurnum* L. extract were higher than the activity of the extract itself. Our findings showed that the combination showed significant activity, higher than zinc oxide nanoparticles and *C. diurnum* L. leaf extract. The combination of the extract and ZnO nanoparticles increases safety and efficiency. The best effectiveness of the combination of alcoholic extract and ZnO-NPs created from this extract might assist in the creation of novel antiviral drugs or as an adjuvant to standard antiviral therapy. It could aid in the treatment of the COVID-19 disease. The current study has promising molecular docking scores and may provide valuable targets for HCOV-229E treatment. The combination of *C. diurnum* L. leaf extract and ZnO nanoparticles should be pharmaceutically formulated and it should be subjected to further investigations to be used as a potential, economic, and ecofriendly antiviral agent.

## Figures and Tables

**Table 1:** Phenolic compounds of *C. diurnum* L. leaf parts identified by HPLC.

No.	Compound	Rt *	RRT *	Area%	Conc (µg/g)
1	Protocatechuic acid	6.751	0.58	1.649	91.02
2	*P*-hydroxy benzoic acid	9.935	0.86	0.1323	6.44
3	Catechin	11.566	1.0	10.656	1425.16
4	Chlorogenic acid	12.194	1.05	0.193	207.46
5	Caffeic acid	13.204	1.14	12.490	13.81
6	Syringic acid	14.698	1.27	5.0717	158.95
7	Vanillic acid	16.279	1.40	4.515	91.14
8	Ferulic acid	20.340	1.76	56.479	1004.68
9	Sinapic acid	21.419	1.85	1.954	38.16
10	Rutin	23.287	2.01	1.139	78.70
11	Rosmarinic acid	29.166	2.52	0.972	90.56
12	Cinnamic acid	35.768	3.09	3.051	25.74
13	Apigenin	39.371	2.97	0.808	16.64
14	Kaempferol	41.255	3.567	0.343	8.66
15	Chrysin	53.943	4.66	0.539	5.92

Abbreviations: * RT, retention time; RRT, relative retention time ferulic acid 3.

**Table 2:** Alkaloid components of *C. diurnum* L. leaves extract identified by HPLC.

No.	Compound	RT	RRT *	Area%	Conc (µg/g)
1	Cotinine	4.0	1.0	25.70	10.66
2	Nicotyrine	4.8	1.2	20.39	9.74
3	Solanidine	7.0	1.75	16.69	9.65
4	Nornicotine	8.3	2.08	12.42	8.89
5	Nicotine	10.0	2.5	14.87	8.55

* RRT relative to the cotinine compound.

**Table 3:** Antiviral activity against human coronavirus (229E).

Tested Samples	CC_50_	IC_50_	SI
Leaf extract	237.68	61.15	3.89
ZnO-NPs	145.87	7.01	20.81
Combination (leaf + ZnO-NPs)	179.23	2.41	74.37

N.B: CC_50_ and IC_50_ are expressed as µg/mL.

**Table 4:** Mode of action of antiviral activity.

Mechanism	Sample Concentration (µg/µL)	Virus Control Titer (PFU/mL)	Virus Titer before and Post Treatment (PFU/mL)	Viral Inhibition (%)
Replication	100	7.0 × 10^3^	1.9 × 10^3^	72.8
	50		3.1 × 10^3^	55.7
	25		5.7 × 10^3^	18.5
Adsorption	100	7.0 × 10^3^	5.8 × 10^3^	17.2
	50		6.7 × 10^3^	4.3
	25		7.0 × 10^3^	0
Virucidal	100	7.0 × 10^3^	3.0 × 10^3^	57
	50		4.9 × 10^3^	30
	25		5.8 × 10^3^	17.2

**Table 5:** (DG, interactions) kcal/mol of (natural tested ligands) against the target site of (HCo-V 229E main protease).

Compounds	Docking Score (kcal/mol)	Interactions		
		H. B.	Pi Interactions	Van der Waals
**Crystal Ligand** **(MDP)**	−5.66	His162 and Glu165.	Ala143, Cys144 and His162.	Ala1, Phe139, Ile140, Gly142, Gln163 and His171.
Catechin	−6.52	-	His41 and Cys144.	Thr25, Val26, Leu27, Val42, Asn141, Gly142 and Pro188.
Chlorogenic acid	−7.13	Val 26, Thr47 and Gln187	Ile164	Thr25, Val 26, Leu27, His41, Gly142, Cys144, Glu165, Pro188 and Gln191.
Ferulic acid	−6.00	Val 26.	Pro188.	Thr 25, Val 26, Leu27, His41, Thr 47, Asn141, Gly142, Cys144, Ile164 and Gln187.
Syringic acid	−6.25	Gly142	Leu27, His41 and Cys144.	Thr 25, Val 26, Thr 47, Ile164 and Pro188.
Cotinine	−6.12	Asn141	Thr47 and Cys144	Thr 25, Val 26, Leu27, His41 Asn141, Gly142 and Ala143.
Nicotyrine	−5.75	Cys144	Cys144	Thr 25, Leu27, His41, Thr 47, Asn141, Gly142, Ala143, Cys144 and Glu165.
Solanidine	−6.95	Thr 25	-	Asn24, Thr 25, His41, Ala44, Ser45, Asn46, Thr47 Asn141, Gly142 and Cys144.

## Data Availability

Not applicable.

## Supplementary Materials

The following supporting information can be downloaded at: https://www.mdpi.com/article/10.3390/molecules28010266/s1, Figure S1: UV spectrum of leaf extract (A), ZnO-NPs (B) and combination (c). Figure S2. Zeta potential of combination, Zeta Potential (mV): −25.1. Figure S3: Effect of pH on ZnO-NPs at different time interval.
