# Supplementary material for: Green Synthesized Zinc Oxide Nanoparticles Based on Cestrum diurnum L. of Potential Antiviral Activity against Human Corona 229-E Virus
Source: Molecules. 2022 Dec 28;28(1):266. doi: 10.3390/molecules28010266 (PMC9822259; doi:10.3390/molecules28010266)
Supplement: Supplementary file 1 [file molecules-28-00266-s001.zip › molecules-2072744-supplementary.pdf]

2. Results

2.3.1. UV Analysis:

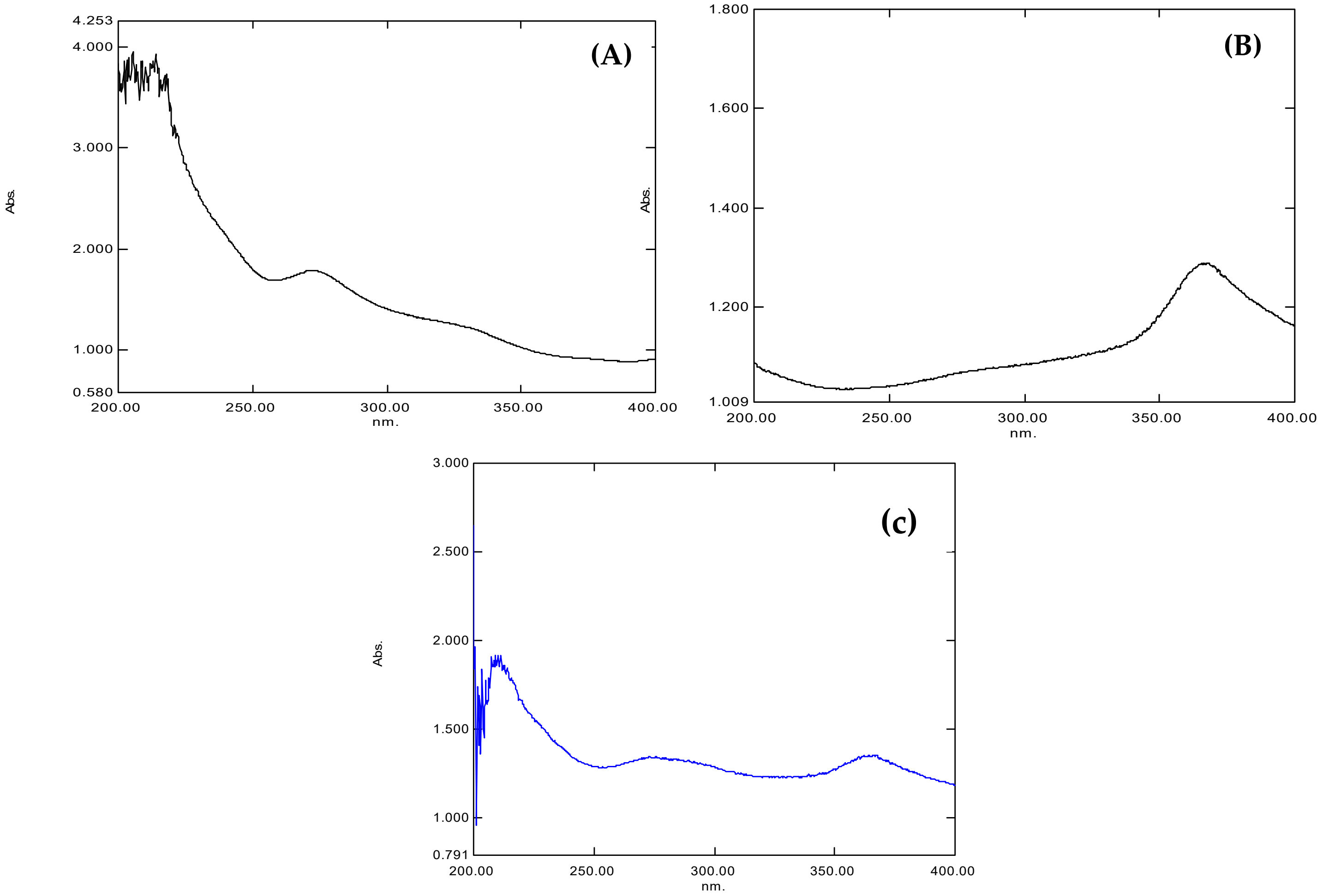

Figure S1. UV spectrum of leaf extract (A), ZnO-NPs (B) and combination (c).

2.3.3. Light Scattering Dynamics and Zeta Potential

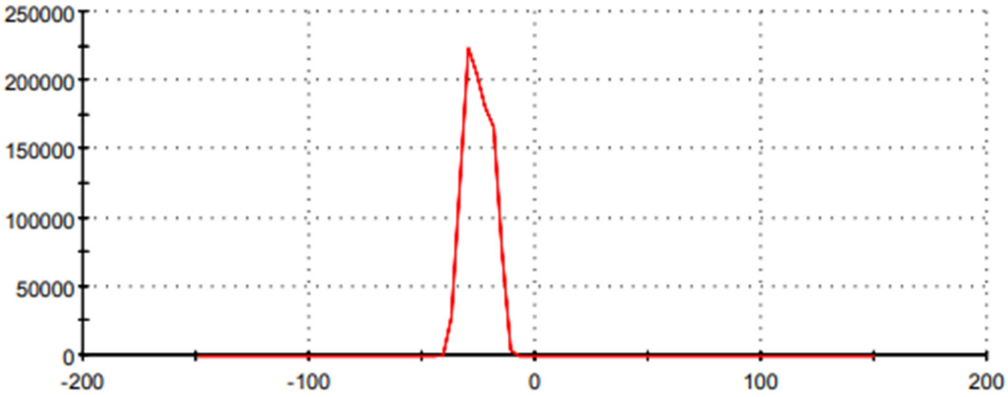

Figure S2. Zeta potential of combination, Zeta Potential: --25.1 mV.

2.6. Chemical Stability Analysis

2.6.1. Effect of pH on ZnO-NPs stability

The synthesized ZnO NPs stability are affected by the variation of pH. The positive charges on the surface of synthesized ZnO NPs can cause strong repulsion among the particles and may prevent them from aggregation.

At pH 3 (Figure 3S A), it was observed an increase in the absorbance at different time intervals. This issue can be interpretated due to decomposition of the capping agent (phenolics and alkaloids metabolites of plant extract) leading to liberation or de-agglomeration of capped nanoparticles.

At pH 5 (Figure 3S B), it was observed an increase in the absorbance at different time intervals but it start to decrease gradually after 4 hrs.

On the other hand at pH 9, it was observed an increase in the absorbance at 2, 4 and 8 hrs and the absorbance decreased after 24 hr. This issue can be result of decomposition of capping agent followed by attractive force hydroxyl ions which carry negative charge towards positive charge of zinc ion leading to aggregation of the nanoparticles.

These findings prove the stability of the prepared zinc oxide nanoparticles.

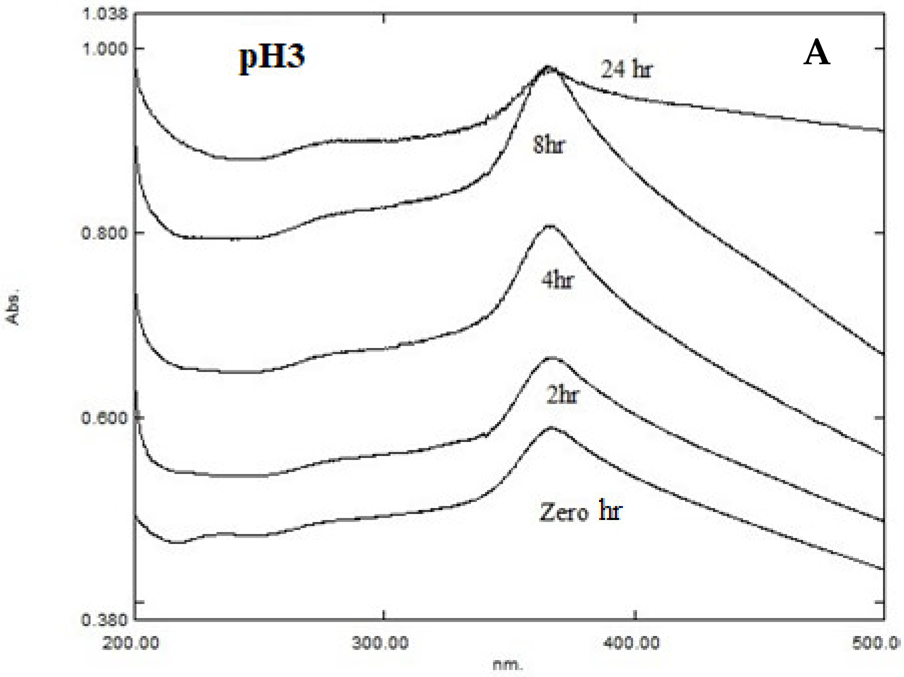

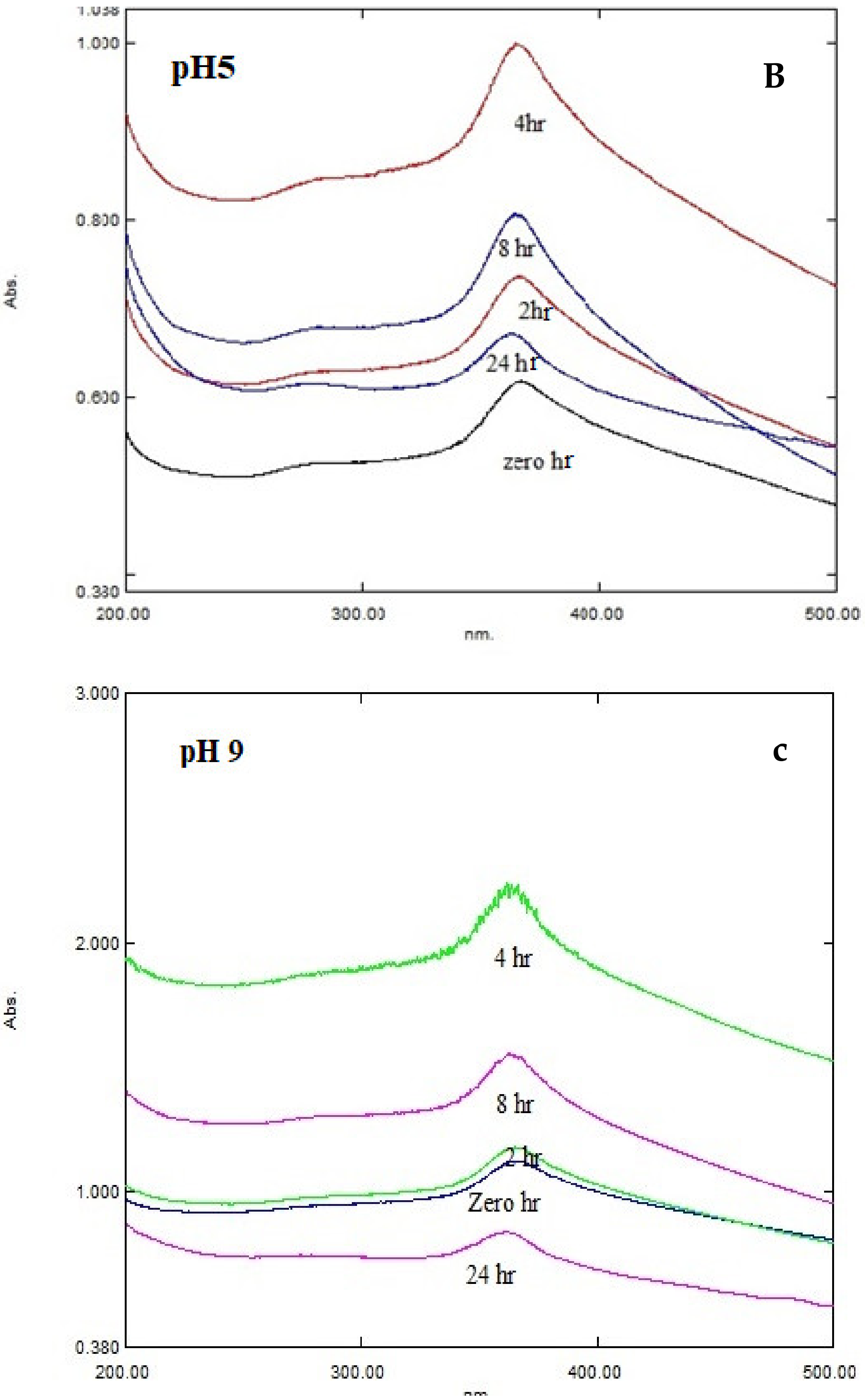

Figure S3. Effect of pH on ZnO-NPs at different time interval

3. Materials and Methods

3.6.1. Chemical Stability Analysis

Preliminary experiments were performed on an Shimadzu-1601 UV–Vis spectrophotometer to investigate the chemical stability of ZnO-NPs at different pH values according to method described by Rónavári et al. 2021 [36].
